# *Seimatosporium chinense*, a Novel Pestalotioid Fungus Associated with Yellow Rose Branch Canker Disease

**DOI:** 10.3390/pathogens13121090

**Published:** 2024-12-10

**Authors:** Haoran Yang, Jing Cheng, Nu Dili, Ning Jiang, Rong Ma

**Affiliations:** 1 College of Forestry and Landscape Architecture, Xinjiang Agricultural University, Urumqi 830052, China; syxy@xjau.edu.cn (H.Y.); dilnur80@163.com (N.D.); 2 Forestry and Grassland Administration of Yili Prefecture, Yining 835000, China; cj17709993198@163.com; 3 Key Laboratory of Biodiversity Conservation of National Forestry and Grassland Administration, Ecology and Nature Conservation Institute, Chinese Academy of Forestry, Beijing 100091, China; n.jiang@caf.ac.cn

**Keywords:** morphology, new species, phylogeny, plant disease, Sporocadaceae

## Abstract

Yellow rose (*Rosa xanthina*) is a common ornamental shrub species widely cultivated in China. However, canker disease symptoms were discovered during our investigations in Beijing and Xinjiang, China. The fungal isolates were obtained from diseased barks and identified using combined methods of morphology and phylogeny based on a partial region of ITS, LSU, *rpb2*, *tef1*, and *tub2* sequences. As a result, a new species of *Seimatosporium* named *S. chinense* was proposed and described herein. The new species is distinguished from its phylogenetic sister species, *S. gracile* and *S. nonappendiculatum*, by conidial characters. The present study improves the species concept in *Seimatosporium* and provides fundamental data for the yellow rose canker disease control in the future.

## 1. Introduction

The genus *Rosa* is famous for its beautiful flowers, of which yellow rose (*R. xanthina*) is native to central China and distributed in open slopes and scrubby areas [1,2,3]. Meanwhile, *R. xanthina* is widely cultivated as an ornamental plant in China [1,2,3]. Recent publications have reported several plant diseases affecting *Rosa* hosts. For example, *Xanthomonas alfalfae* subsp. *rosa* causes bacterial leaf spot disease [4], *Podosphaera pannosa* is responsible for rose powdery mildew [5], *Diaporthe rosiphthora* leads to rose dieback [6], and *Neopestalotiopsis rosicola* causes branch canker of *Rosa chinensis* in China [7].

Fungal species within the family Sporocadaceae are commonly referred to as pestalotioid fungi, characterized by multi-septate, fusiform conidia typically with conidial appendages [8,9,10,11,12]. The most common genus, *Pestalotiopsis*, is frequently associated with plant diseases [13,14,15,16]. Other pestalotioid fungi, previously classified in genera such as *Monochaetia*, *Seimatosporium*, and *Seiridium*, have been reclassified based on a combination of molecular phylogeny and morphological features [8,17].

The genus *Seimatosporium* was originally established based on its type species, *S. rosae* [18]. However, its generic concept has undergone multiple revisions since its inception, with up to 25 synonyms currently recognized [8]. Members of this genus are now defined by acervular or pycnidioid conidiomata, colorless, smooth, septate, and branched conidiophores, which may be discrete or integrated, and subcylindrical, cylindrical, ampulliform, or lageniform annellidic conidiogenous cells. The conidia are fusoid, ellipsoid, ovoid, or clavate, with brown median cells and an appendage [8]. Species within *Seimatosporium* are molecularly distinguished by phylogenetic analysis based on combined multi-gene sequences [8,9,18].

Interestingly, species of *Seimatosporium* are known to inhabit two unrelated host genera: *Rosa* (Rosaceae, Rosales) and *Vitis* (Vitaceae, Rhamnales) [9,19,20,21,22]. To date, six species have been identified on *Rosa* hosts, including *S. centrale*, *S. discosioides*, *S. gracile*, *S. nonappendiculatum*, *S. parvum*, *S. pseudorosae*, and *S. rosae* [9], with four of these species reported in China, where *Rosa* hosts are widely distributed [9]. In contrast, ten *Seimatosporium* species have been found on *Vitis* hosts, where they are associated with grapevine trunk diseases [18,20,21].

With over 200 species of *Rosa* widely distributed across China [1], most hosts have yet to be investigated for the presence of *Seimatosporium* endophytes or pathogens. Symptoms of branch canker disease were observed on *Rosa xanthina* branches in Huoying Street, Beijing, and the Urumqi Botanical Garden, Xinjiang. This study aimed to identify the fungal species associated with yellow rose branch canker.

## 2. Materials and Methods

### 2.1. Disease Investigation, Sample Collection and Fungal Isolation

Yellow rose branch canker disease was investigated in Beijing and Xinjiang. The number of plants surveyed and those affected by the disease were recorded, and disease incidence (DI) was calculated as the percentage of diseased plants: DI = (number of diseased plants/number of surveyed plants) × 100%. Cankered branches were cut, placed in paper bags, and sent to the laboratory for fungal isolation and morphological studies.

Diseased branches were surface disinfected by immersion in 75% ethanol for 1 min, then rinsed in sterile water and dried on sterile paper. Tissue samples from the boundary between diseased and healthy bark were cut into 5 × 5 mm fragments and placed on PDA (potato dextrose agar: 200 g potatoes, 20 g dextrose, 20 g agar per L) plates. The plates were incubated at 25 °C to promote the growth of fungal hyphae. After 24 h, hyphae were grown from the bark tissues on the plates, and hyphal tips were cut and removed to the new PDA plates under a stereomicroscope to obtain clean cultures. The specimens were deposited in the herbarium of the Chinese Academy of Forestry (CAF) and the ex-type strain in the China Forestry Culture Collection Center (CFCC).

### 2.2. Morphological Observation

Morphological studies of the fungal species in this study were based on the conidiomata naturally formed on the host branches and cultures grown on PDA, OA (oatmeal agar, 60 g oatmeal, 12.5 g agar per L), and SNA (synthetic nutrient deficient agar, 0.2 g glucose, 0.2 g sucrose, 1 g potassium dihydrogen phosphate, 1 g potassium nitrate, 0.25 g magnesium sulfate anhydrous, 0.5 g potassium chloride, and 14 g agar per L). The conidiomata were observed and photographed using a dissecting microscope (Discovery V8; Zeiss, Oberkochen, Germany). Microscope slides of conidiogenous cells and conidia were prepared in tap water, and the slides were examined and photographed using a Nikon Eclipse 80i microscope (Nikon, Tokyo, Japan) equipped with a Nikon digital sight DS-Ri2 camera, using differential interference contrast (DIC) illumination.

### 2.3. Molecular Studies

The genomic DNA was extracted from colonies grown on cellophane-covered PDA using a CTAB (cetyltrimethylammonium bromide) method [23]. Five loci, including ITS (the 5.8S nuclear ribosomal DNA gene with the two flanking internally transcribed spacer regions), LSU (the large subunit of the nrDNA), *rpb2* (DNA-directed RNA polymerase II second largest subunit), *tef1* (the translation elongation factor 1-alpha gene), and *tub2* (the β-tubulin gene), were amplified using primer pairs ITS1/ITS4, LR0R/LR5, rpb2-5f2/rpb2-7cr, EF1-728F/EF2, and Bt2a/Bt2b, respectively [24,25,26,27,28]. Then, PCR was conducted following the method recorded by Razaghi et al. [17]. All amplified PCR products were estimated visually using 1.4% agarose gels stained with ethidium bromide. Then, the PCR-positive products were sent to Sangon Biotech (Shanghai) Co., Ltd. (Beijing, China) for sequencing. Sequences from the present and recent publications [8,9,17] were listed in Table 1 for phylogenetic analyses.

Sequences from five loci (ITS, LSU, *rpb2*, *tef1*, and *tub2*) were aligned using MAFFT v.7 (http://mafft.cbrc.jp/alignment/server/index.html, accessed on 10 July 2024) and manually edited in MEGA v.7.0.21. Phylogenetic inferences of the concatenated alignments were performed using Maximum Likelihood (ML) and Bayesian Analysis (BA). ML and BA were implemented through the CIPRES Science Gateway portal (https://www.phylo.org/, accessed on 10 July 2024) using RAxML-HPC BlackBox v.8.2.10 and MrBayes v.3.2.6, respectively [29,30,31]. The parameters for the phylogenetic analyses followed those described by Razaghi et al. [17]. The resulting phylogram was plotted using FigTree v.1.4.2.

## 3. Results

### 3.1. Disease Symptoms and Incidence

**Figure 1:**
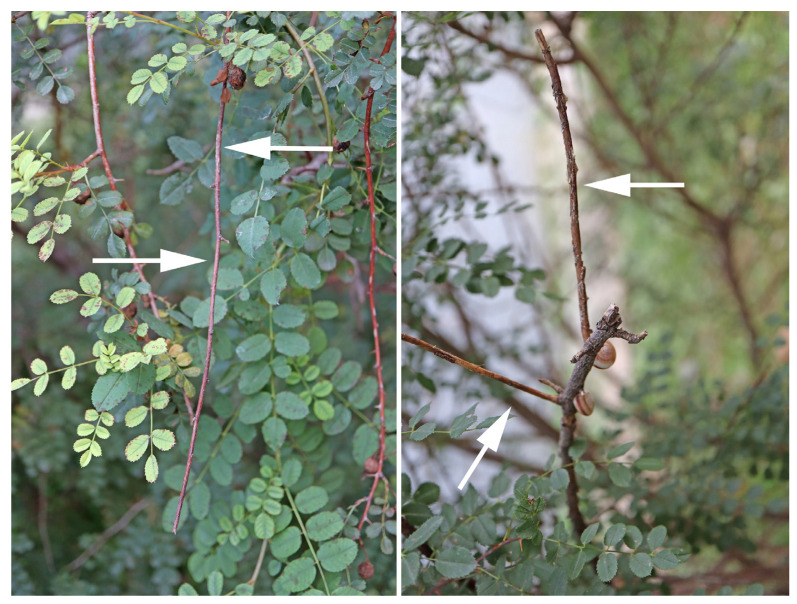
Symptoms of yellow rose canker disease: arrows show diseased branches.

Symptoms of canker disease were observed on the branches of *Rosa xanthina* in Huoying Street, Beijing, and the Urumqi Botanical Garden, Xinjiang (Figure 1). Affected branches displayed sunken surface bark and discolored inner bark beneath the cankers. Over time, visible fruiting bodies developed on the diseased branches. The disease incidence was 1.75% in Huoying Street and 5% in the Urumqi Botanical Garden.

### 3.2. Phylogeny

**Figure 2:**
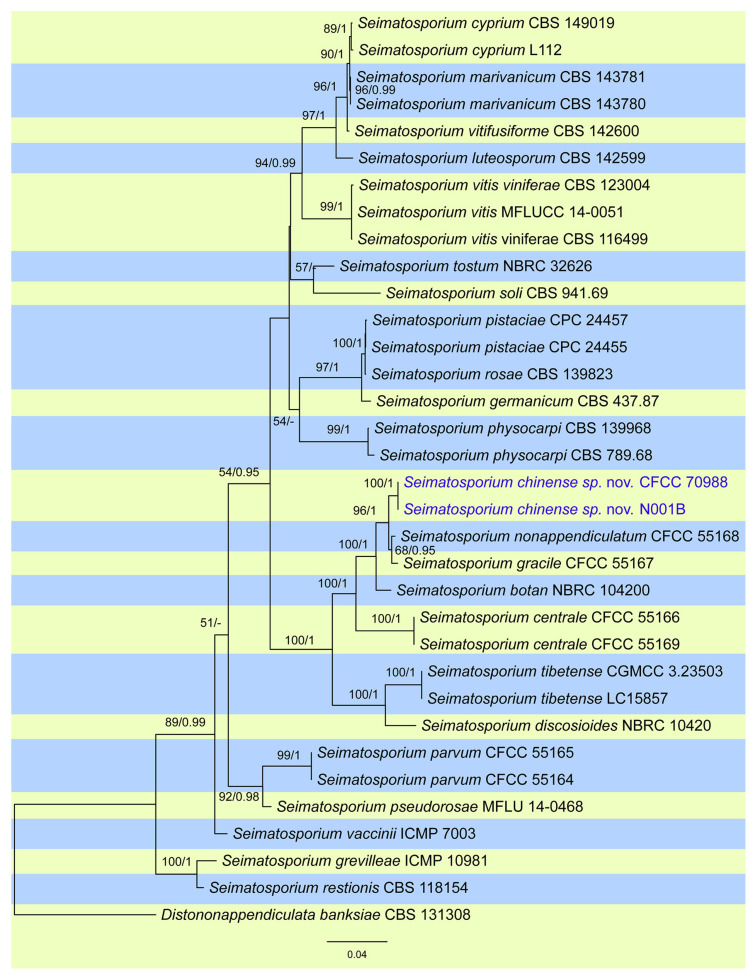
Phylogram of *Seimatosporium* resulting from a maximum likelihood analysis based on a combined matrix of ITS, LSU, *rpb2*, *tef1*, and *tub2* loci. Numbers above the branches indicate ML bootstrap values (left, ML BS ≥ 50%) and Bayesian posterior probabilities (right, BPP ≥ 0.9). Isolates from the present study are marked in blue.

The DNA sequence dataset of combined ITS, LSU, *rpb2*, *tef1*, and *tub2* loci was used to construct the phylogram of *Seimatosporium* and identify the strains obtained from the current study. The concatenated alignment contains 3907 characters, including alignment gaps (512 for ITS, 1186 for LSU, 1070 for *rpb2*, 500 for *tef1*, and 639 for *tub2*). The final ML optimization likelihood value of the best RAxML tree was −13632.66, and the matrix had 997 distinct alignment patterns, with 34.22% undetermined characters or gaps. Estimated base frequencies were as follows: A = 0.252125, C = 0.249282, G = 0.246908, and T = 0.251685; substitution rates AC = 1.725640, AG = 4.400041, AT = 1.252508, CG = 1.283665, CT = 8.194629, and GT = 1.0; and gamma distribution shape parameter α = 0. 144615. The topology resulted from RAxML, and Bayesian analyses were congruent. CFCC 70988 and N001B from the current study formed an independent clade (ML/BI = 100/1) and are phylogenetically close to *S*. *gracile* and *S*. *nonappendiculatum* (Figure 2).

### 3.3. Description of the New Species

*Seimatosporium chinense* Ning Jiang, sp. nov.

MycoBank MB855657

**Figure 3:**
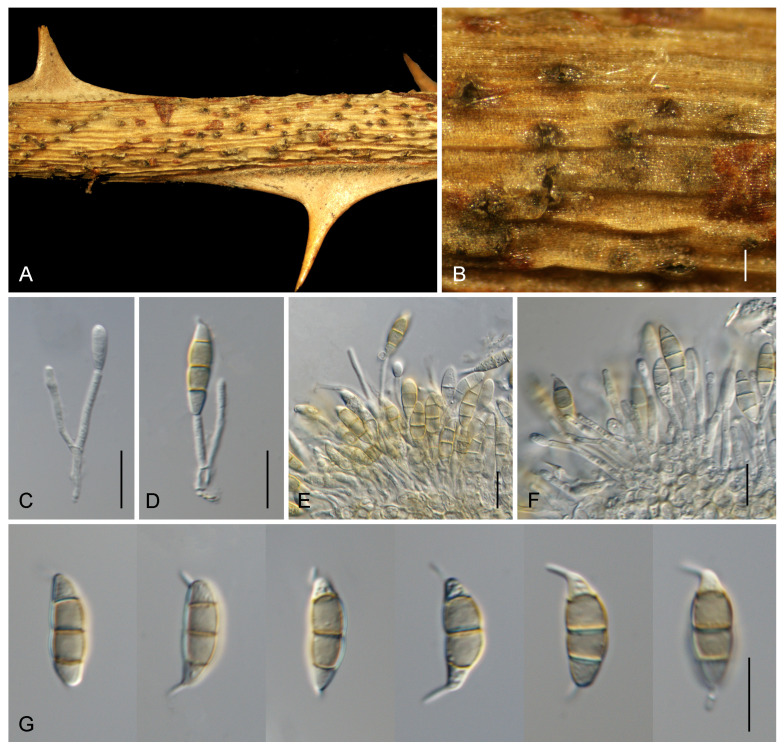
*Seimatosporium chinense*. (**A**) Disease symptoms. (**B**) Appearance of conidiomata on host substrate. (**C**–**F**) Conidiogenous cells with attached conidia. (**G**) Conidia.—Scale bars: (**B**) = 200 μm; (**C**–**G**) = 10 μm.

**Figure 4:**
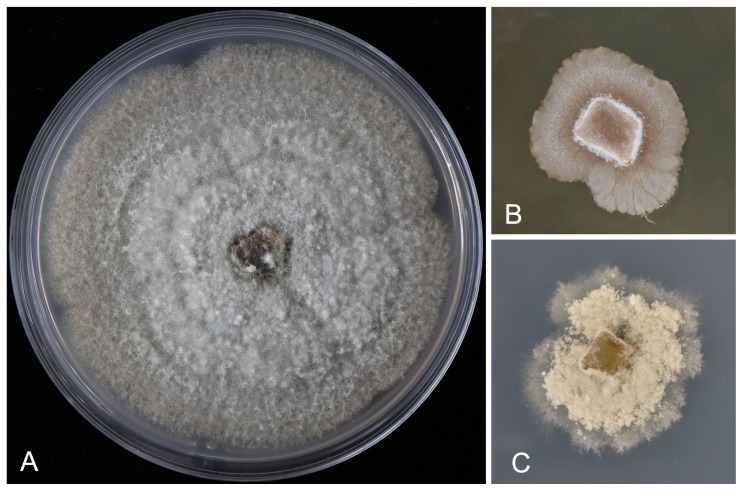
Colonies of *Seimatosporium chinense* grown on PDA (**A**), OA (**B**), and SNA (**C**) after 20 days.

Figure 3 and Figure 4

Etymology. Named after the country, China.

Teleomorph not observed. Anamorph: Acervular conidiomata solitary to gregarious, semi-immersed, pulvinate. Conidiophores branched at the base, hyaline. Conidiogenous cells discrete or integrated, mostly cylindrical to subcylindrical, or sometimes subglobose, hyaline, smooth, thick-walled, (8.5–)9.5–18(–24) × (1.5–)2–3.5(–4) μm. Conidia straight to slightly curved, fusoid, 3-septate, mostly constricted at the septa, transverse septa fairly thick, smooth-walled, (15–)15.5–17.5(–18.5) × (4–)5.5–6.5(–7) μm; basal cell obconic with a truncate base, hyaline to pale brown, 3.5–4 μm long; middle cells cylindrical, brown, second cell from base 4–6 μm, third cell from base 4–6 μm; apical cell conical, hyaline to pale brown, 3.5–4 μm; apical appendage single, unbranched, centric, occasionally excentric, 2.5–4.5 μm long; basal appendage single, unbranched, centric, occasionally excentric, 2–5 μm long; length/width ratio = 2.6–3.1.

Culture characteristics. On PDA, colonies are flat with an entire edge concentric, showing moderate aerial mycelium; initially white, becoming fawn, sterile, and reaching 90 mm diam after 15 d at 25 °C. On OA, colonies are flat with undulate margin and sparse aerial mycelium, rosy to pale luteous, sterile, and reaching 30 mm diam after 30 d at 25 °C. On SNA, colonies are flat with an undulated margin and flocculent aerial mycelium, white, and sterile, reaching 35 mm diam after 30 d at 25 °C (Figure 4).

Materials examined. China, Beijing City, Changping District, Huoying Street, on cankered branches of *Rosa xanthina*, 5 July 2023, Ning Jiang (**holotype** CAF800098, **ex-type culture** CFCC 70988); Xinjiang Uygur Autonomous Region, Urumqi City, Gaoxin District, Ergong Street, Urumqi Botanical Garden, on branches of *Rosa xanthina*, 8 May 2024, Rong Ma (culture N001B).

Notes. *Seimatosporium chinense* from *Rosa xanthina* is phylogenetically close to *S*. *gracile* from *R*. *xanthina* and *S*. *nonappendiculatum* from *R. laevigata* (Figure 2). Morphologically, *S. chinense* differs from *S*. *gracile* and *S*. *nonappendiculatum* in conidial characters [(15–)15.5–17.5(–18.5) × (4–)5.5–6.5(–7) μm in *S. chinense* **vs.** (13.5–)14.5–19.5(–20) × 2.5–3(–3.5) μm in *S*. *gracile* **vs.** (17–)18–23.5(–24) × (5–)5.5–7 μm in *S*. *nonappendiculatum*] [9]. Briefly, *S. chinense* has obviously longer conidia than *S*. *gracile* and a bit shorter conidia than *S*. *nonappendiculatum*. In addition, *S. chinense* can be distinguished from *S*. *nonappendiculatum* by forming an apical conidial appendage, which is absent in *S*. *nonappendiculatum* [9].

## 4. Discussion

*Seimatosporium* is a pestalotioid genus commonly inhabiting hosts in the *Rosa* and *Vitis* host genera as endophytes, pathogens, or saprophytes [9,18,20,32]. Grapevine trunk diseases caused by members of *Seimatosporium* have been well studied, revealing a diverse array of species and their varying levels of aggressiveness [18,20,21,22]. However, pathogenicity studies on *Rosa* spp. remain limited. Peng et al. [9] examined Chinese pestalotioid species associated with *Rosa* hosts, focusing on species diversity, yet the pathogenicity of these fungi inhabiting *Rosa* has not been evaluated.

The species concept of *Seimatosporium* has changed significantly since molecular phylogeny has been applied to delineate species [17,18,19]. According to records on the Index Fungorum website (https://www.indexfungorum.org/Names/Names.asp, accessed on 15 July 2024), up to 111 epithets have been documented. However, members of this genus have frequently been reclassified, many of which become synonyms [17,18]. Currently, 23 species are accepted within this genus, supported by morphological descriptions and available sequence data (Table 1, Figure 2).

In this study, we propose *Seimatosporium chinense* sp. nov. from *Rosa xanthina* in China, which is phylogenetically related to *S. gracile* from *R. xanthina* and *S. nonappendiculatum* from *R. laevigata* (Figure 2). *S. chinense* shares the same host and distribution as *S. gracile* but has distinctly wider conidia, which serves as a key distinguishing feature among pestalotioid species [9]. In contrast, *S. nonappendiculatum* is distinguished from its two sister species by the absence of apical appendages [9]. Thus, these three phylogenetically close species are easily distinguished from each other by their conidial morphology, including differences in shape and size.

In ornamental shrubs, branch diseases are often overlooked due to regular trimming of dead branches. While removing diseased branches can effectively mitigate disease development, the wounds created during trimming can facilitate host infection. Therefore, more attention is needed to manage this disease, which was previously reported in a different species, and control measures are necessary when it emerges.

## Figures and Tables

**Table 1:** Strains and GenBank accession numbers used in this study.

Species	Strain	Host	Country	GenBank Accession Number				
				ITS	LSU	*rpb2*	*tef1*	*tub2*
* Distononappendiculata banksiae *	CBS 131308 *	* Banksia marginata * (Proteaceae)	Australia	JQ044422	AB593705	NA	NA	MH554670
* Seimatosporium botan *	NBRC 104200 *	* Paeonia suffruticosa * (Paeoniaceae)	Japan	AB594799	AB593731	NA	NA	LC047770
*Seimatosporium centrale*	CFCC 55166 *	*Rosa chinensis* (Rosaceae)	China	OK560629	OK560399	ON055447	OM986918	OM301641
* Seimatosporium centrale *	CFCC 55169	* Rosa chinensis * (Rosaceae)	China	OK560632	OK560402	ON055450	OM986921	OM301644
* Seimatosporium chinense *	CFCC 70988 *	* Rosa xanthina * (Rosaceae)	China	PQ279535	PQ279531	PQ283812	PQ287323	PQ287325
* Seimatosporium chinense *	N001A	* Rosa xanthina * (Rosaceae)	China	PQ279536	PQ279532	PQ283813	PQ287324	PQ287326
*Seimatosporium cyprium*	CBS 149019 *	* Vitis vinifera * (Vitaceae)	Cyprus	ON680684	ON705769	NA	ON863790	ON695856
*Seimatosporium cyprium*	L112	* Vitis vinifera * (Vitaceae)	Cyprus	ON695889	ON692404	NA	ON863791	ON695848
*Seimatosporium discosioides*	NBRC 10420	*Punica granatum* (Lythraceae)	Japan	AB594800	AB593732	NA	NA	LC047771
*Seimatosporium germanicum*	CBS 437.87 *	NA	Germany	MH554047	MH554259	MH554957	MH554482	MH554723
*Seimatosporium gracile*	CFCC 55167 *	* Rosa xanthina * (Rosaceae)	China	OK560638	OK560408	ON055456	OM986927	OM301650
*Seimatosporium grevilleae*	ICMP 10981	* Protea * sp. (Proteaceae)	South Africa	AF405304	AF382372	NA	NA	NA
*Seimatosporium luteosporum*	CBS 142599 *	* Vitis vinifera * (Vitaceae)	USA	KY706284	KY706309	NA	KY706334	KY706259
*Seimatosporium marivanicum*	CBS 143781 *	* Vitis vinifera * (Vitaceae)	Iran	MW361952	MW361960	NA	MW375358	MW375352
*Seimatosporium marivanicum*	CBS 143780	* Vitis vinifera * (Vitaceae)	Iran	MW361951	MW361959	NA	MW375357	MW375351
*Seimatosporium nonappendiculatum*	CFCC 55168 *	* Rosa laevigata * (Rosaceae)	China	OK560657	OK560427	ON055475	OM986946	OM301669
*Seimatosporium parvum*	CFCC 55164 *	* Rosa spinosissima * (Rosaceae)	China	OK560647	OK560417	ON055465	OM986936	OM301659
*Seimatosporium parvum*	CFCC 55165	* Rosa helenae * (Rosaceae)	China	OK560653	OK560423	ON055471	OM986942	OM301665
*Seimatosporium physocarpi*	CBS 139968 *	* Physocarpus opulifolius * (Rosaceae)	Russia	KT198722	KT198723	MH554917	MH554434	MH554676
*Seimatosporium physocarpi*	CBS 789.68	* Physocarpus amurensis * (Rosaceae)	Netherlands	MH554066	MH554278	MH554979	MH554502	MH554742
*Seimatosporium pistaciae*	CPC 24455 *	*Pistacia vera* (Anacardiaceae)	Iran	KP004463	KP004491	MH554915	MH554432	MH554674
*Seimatosporium pistaciae*	CPC 24457	* Pistacia vera * (Anacardiaceae)	Iran	MH554126	MH554331	MH555035	MH554561	MH554799
*Seimatosporium pseudorosae*	MFLU 14-0468 *	* Rosa villosa * (Rosaceae)	Italy	NA	KU359035	NA	NA	NA
*Seimatosporium restionis*	CBS 118154 *	* Restio filiformis * (Restionaceae)	South Africa	DQ278922	DQ278924	NA	NA	NA
*Seimatosporium rosae*	CBS 139823 *	* Rosa kalmiussica * (Rosaceae)	Russia	KT198726	KT198727	LT853153	LT853203	LT853253
*Seimatosporium soli*	CBS 941.69 *	Forest soil	Denmark	MH554071	MH554282	MH554983	MH554507	NA
*Seimatosporium tibetense*	CGMCC 3.23503 *	NA	China	OR247936	OR247954	OR380975	OR361511	OR381084
*Seimatosporium tibetense*	LC15857	NA	China	OR247937	OR247955	OR380976	OR361512	OR381085
*Seimatosporium tostum*	NBRC 32626	NA	Unknown	AB594795	AB593727	NA	NA	NA
*Seimatosporium vaccinii*	ICMP 7003	* Vaccinium ashei * (Ericaceae)	New Zealand	NA	AF382374	NA	NA	NA
*Seimatosporium vitifusiforme*	CBS 142600 *	* Vitis vinifera * (Vitaceae)	USA	KY706296	KY706321	NA	KY706346	KY706271
*Seimatosporium vitis*	MFLUCC 14-0051 *	* Vitis vinifera * (Vitaceae)	Italy	KR920363	KR920362	NA	NA	NA
*Seimatosporium vitis-viniferae*	CBS 123004 *	* Vitis vinifera * (Vitaceae)	Spain	MH553992	MH554211	MH554901	MH554418	MH554660
*Seimatosporium vitis-viniferae*	CBS 116499	* Vitis vinifera * (Vitaceae)	Iran	MH553984	MH554201	MH554884	MH554402	MH554643

Notes: NA, not applicable. * ex-type strains.

## Data Availability

The sequence data obtained from the present study has been uploaded in NCBI GenBank with accession numbers listed in Table 1.
